# Lymphocyte Activation Gene 3 Single-Nucleotide Polymorphisms in Bone Marrow Failure Diseases

**DOI:** 10.1155/2022/3528598

**Published:** 2022-02-27

**Authors:** Yingying Sun, Qiuying Cao, Xiaoyu Zhao, Chunyan Liu, Zonghong Shao

**Affiliations:** Department of Hematology, Tianjin Medical University General Hospital, Tianjin, China

## Abstract

**Introduction:**

Lymphocyte activation gene 3 (LAG3) is an inhibitory checkpoint protein expressed on activated T effector, T regulatory, and natural killer cells. The main function of LAG3 is the regulation of immune homeostasis. Several studies have suggested its role in malignant and autoimmune diseases. The objective of this study was to explore the association between *LAG3* single-nucleotide polymorphisms (SNPs) and bone marrow failure diseases.

**Methods:**

Sixty-two patients newly diagnosed with bone marrow failure diseases in the Hematology Department of Tianjin Medical University General Hospital between January 2019 and December 2020 and 16 healthy controls were enrolled in this study. SNPs in *LAG3* were investigated by performing Sanger sequencing, and the association of the detected SNPs with bone marrow failure diseases was analyzed.

**Results:**

Eleven SNPs were identified. Among them, the frequency of LAG3 rs1941928301 (C>T) was statistically different among the groups (*P* = 0.013). It was higher in the myelodysplastic syndrome (MDS) group than that in the severe aplastic anemia (SAA) group (*P* = 0.004) and that in the healthy control group (*P* = 0.009).

**Conclusions:**

LAG3 rs1941928301 (C>T) might be associated with a higher risk of MDS. The detected *LAG3* SNPs have no apparent effect on susceptibility to SAA and immune-related pancytopenia (IRP).

## 1. Introduction

Bone marrow failure diseases are common in patients admitted in the department of hematology of Tianjin Medical University General Hospital. The bone marrow failure diseases commonly observed are severe aplastic anemia (SAA), mainly caused by the hyperfunction of cytotoxic T lymphocytes; immune-related pancytopenia (IRP), mainly caused by abnormal antibodies produced by B lymphocytes to attack hematopoietic cells; and myelodysplastic syndrome (MDS), a clonal neoplastic disease characterized by the increased risk of transforming into acute myeloid leukemia (AML). As these diseases have different pathogenic mechanisms, the treatment principles of each disease are different. An accurate diagnosis of these diseases is crucial. In fact, the differential diagnosis of bone marrow diseases is challenging, because the diseases evolve over time and in different care settings, and in many cases, the clinical presentation of these diseases is atypical. Therefore, mechanisms underlying the pathophysiology of these diseases need to be elucidated, and the discovery of specific diagnostic indicators may help in early diagnosis and improvement in patient symptoms and disease prognosis.

Lymphocyte activation gene 3 (LAG3) is an inhibitory checkpoint protein that is expressed on activated T effector cells, T regulatory cells, and natural killer cells. The main function of LAG3 is the regulation of immune homeostasis [1]. In our previous study, we observed a reduction in LAG3 expression in patients with SAA [2] and an increase in LAG3 expression in patients with MDS [3], suggesting that abnormal LAG3 expression may lead to the maladjustment of T-cell immunity, thus causing subsequent changes. However, genetic factors involved in the regulation of LAG3 expression are unknown.

In this study, we performed Sanger sequencing to detect the occurrence of mutations in *LAG3* in bone marrow failure diseases. We aimed to explore the association between *LAG3* single-nucleotide polymorphisms (SNPs) and bone marrow failure diseases.

## 2. Materials and Methods

### 2.1. Subjects

A total of 62 patients newly diagnosed with bone marrow failure diseases in the Hematology Department of Tianjin Medical University General Hospital between January 2019 and December 2020 and 16 healthy controls (HCs) were enrolled in this study. The 62 patients were further categorized as patients with SAA (*n* = 16), patients with IRP (*n* = 25), and patients with MDS (*n* = 21). The diagnosis of SAA was performed in compliance with the 2016 International AA Study Group criteria [4]. The diagnosis of MDS was performed in compliance with the 2016 WHO criteria [5]. IRP is defined as hemocytopenia caused by bone marrow hematopoietic cell-specific antibodies [6]. The characteristics of the enrolled subjects are summarized in Table 1. There was no significant difference in sex and age composition among the four groups (SAA, IRP, MDS, and HCs) (*P* > 0.05). The study was approved by the ethics committee of Tianjin Medical University General Hospital (Registered number: IRB2020-KY-375). Informed written consent was obtained from all participants or their guardians. The study was performed in accordance with the Declaration of Helsinki.

### 2.2. DNA Extraction

Peripheral blood samples from all study participants were collected in ethylene diamine tetraacetic acid (EDTA) anticoagulant tubes. Red blood cells were lysed using Red Blood Cell Lysis Buffer (Solarbio, Beijing, China). Genomic DNA was extracted from the peripheral blood cells using the TIANamp Blood DNA Kit (TIANGEN, Beijing, China) according to the manufacturer's instructions. The DNA products were stored at −20°C until further use.

### 2.3. DNA Sequencing

The ABI3730 Sequencing Scanner was used to analyze SNPs in *LAG3* in the genomic DNA of 78 study participants. *LAG3* has eight exons. Seven pairs of primers were designed, among which exon 6 was sequenced in the reverse direction, and the others were sequenced in the forward direction (see Table 2). The cycling protocol used was 96°C for 1 min, followed by 25 amplification cycles (96°C for 10 s, 50°C for 5 s, and 60°C for 4 min), and 4°C for 10 s to terminate the reaction. The results were processed using Sequencing Analysis v5.2 and SeqScape v2.5 software. ANNOVAR was used for SNP annotation.

### 2.4. Statistical Analysis

The statistical analysis software SPSS 26 was used for data analysis. The one-way analysis of variance was used to evaluate the differences among continuous variables. The sex composition was determined using the chi-square test. Fisher's exact test was used for the analysis of SNPs among groups, and a post hoc least significance difference (LSD) test was used for multiple comparisons. Results with *P* < 0.05 were considered statistically significant.

## 3. Results

Eleven mutations were identified using Sanger sequencing, and all of them were SNPs. Their positions, reference allele, altered allele, function of reference genotypes, and frequency of each SNP in different groups are summarized in Table 3. Seven SNPs were identified in patients with MDS, 6 in patients with IRP, and 3 in patients with SAA.

All SNPs were filtered using 1000 genomes to identify the diversity sites among individuals. Three mutations with a frequency higher than 0.01 were filtered after this procedure, among which SNP6881835 was previously labeled as NM_002286:c.-184G>A and rs7342333, and its frequency in 1000 genomes was 0.049321; SNP6883700 was previously labeled as rs2365095, and its frequency in 1000 genomes was 0.388778; SNP6887020 was previously labeled as rs870849, and its frequency in 1000 genomes was 0.669329. Besides, in our study, the frequency of these three SNPs showed no statistical difference among the groups.

SNP 6884554, labelled as NM_002286:exon5:c.T897C:p.T299T, is located in the exonic region but does not affect the amino acid coded by the gene. It is a synonymous mutation and has no effect on the translated protein; therefore, it was filtered out. Furthermore, there was no statistical difference in frequency among the four groups.

The remaining seven SNPs were subject to correlation analysis. Among them, only the frequency of SNP at position 6887343(rs1941928301C>T) was statistically different among the groups (*P* = 0.013). It was higher in the MDS group than that in the SAA group (*P* = 0.004) and HC group (*P* = 0.009). SNP6887343 (C>T) was located in the intronic region, and the frequency of the minor allele (T) was 11.9% in patients with MDS, 4.0% in patients with IRP, and 0% in patients with SAA and HCs. Genotypic distribution of rs1941928301 in *LAG3* (TT, TC, and CC) among the four groups showed no significant differences.

## 4. Discussion

Various pathogenic factors might be associated with bone marrow failure. SAA is mainly caused by abnormal cellular immunity and can be treated using antithymocyte globulin (ATG) and cyclosporine. IRP is induced by specific antibodies produced by dysfunctional B lymphocytes and can be treated using glucocorticoids and rituximab. MDS usually involves the expansion of myeloid malignant clones and T-lymphocyte exhaustion, which often require demethylation or chemotherapy. These diseases have different etiologies, treatments, and prognoses.

In recent years, exploration of autoimmune disease pathogenesis and tumor immunity has showed the critical role of immune checkpoint molecules in the pathophysiology and treatment of autoimmune diseases and cancer. Among them, LAG3 is a negative regulator of the immune system, which negatively regulates the proliferation and activation of effector T cells and maintains the inhibitory activity of regulatory T cells. Several studies have reported the role of LAG3 in the immunotherapy of neoplastic diseases, and some studies have also reported that LAG3 deficiency is involved in the pathogenesis of autoimmune diseases [7–10]. Previously, we reported the difference in LAG3 expression between T cells of patients with SAA and those of patients with MDS, suggesting that LAG3 may be involved in the occurrence of these two diseases. However, studies on genetic mutations in *LAG3* in patients with bone marrow failure diseases are still limited. In this study, we investigated the mutations in patients with bone marrow failure diseases using Sanger sequencing to identify association of genetic variations in *LAG3* with these diseases.

*LAG3* is located on chromosome 12p13. Eleven SNP sites were discovered in the study. Among them, SNPs at positions 6881748 (rs546638559), 6881835 (rs7342333), and 6881946 (rs1229251092) are located in the untranslated region (UTR)5. UTR5 is the untranslated part at the 5′ end of the mRNA. Genetic variants in this position may affect mRNA transport, stability, and translation. However, these three SNPs have not been reported to be associated with any other disease susceptibility, and there was also no significant difference among the four groups in our study.

Two SNP sites were found in the exons of *LAG3*. Among them, SNP at position 6884554 was labelled as NM_002286:exon5:c.T897C:p.T299T. It is located in the fifth exon and is a synonymous SNP. SNP at position 6887020 (rs870849) is located in the seventh exonic region of *LAG3*. This variation is a nonsynonymous mutation, labelled as NM_002286:exon7:c.T1364C:p.I455T. Previous studies have shown that the T allele of LAG3 rs870849 is a protective factor for ITP severity [11], while it is unlikely to affect type 1 diabetes mellitus susceptibility [12]. In our study, there was no significant difference among the four groups, indicating that rs870849 did not affect the susceptibility of bone marrow failure diseases.

The other six SNP sites are located in the intron region. SNPs 6883680 (rs1941881771) and 6883700 (rs2365095) are located in the third intronic region. LAG3 rs2365095 has been found to be associated with multiple sclerosis in the Jordanian Arab population [13]. SNP 6886722 (rs199912414) is located in the sixth intronic region, and no association with disease was found in the past. In this study, these three SNPs showed no difference among the four groups. SNPs at position 6887343, 6887348, and 6887383 are located in the seventh intronic region and have not previously been linked to any disease. In our experiment, the frequency of SNP 6887343(C>T), which was labelled as rs1941928301, was significantly higher in patients with MDS than in patients with SAA and HCs. We concluded that the higher risk of MDS occurrence is associated with allele T. Our study showed the genetic association between LAG3 rs1941928301 and MDS.

The pathophysiology of MDS involves genetic and epigenetic alterations leading to the amplification of myeloid blasts. A plethora of candidate genes correlated with MDS susceptibility have been previously identified. Studies have focused on genes involved in cancer-related pathways, including methylation regulation and clonal amplification [14, 15]. However, treatments that target these pathways, such as induction chemotherapy and demethylation, have a low efficacy. Therefore, several researchers have explored other alternatives with the goal to further delay progression and improve survival. The dysfunction of immune system has also been shown to participate in MDS pathogenesis and progression, especially cellular immunity defects, which are mainly manifested as reduced number and function of CD8^+^T cells, imbalance of T helper (Th)1/Th2 ratio, and increased proportion of regulatory T cells [16]. Ideally, effector T cells are activated by antigen presenting cell- (APC-) presented antigens and receive primary and secondary signals, and then clear tumor antigens. However, tumor cells can use various mechanisms to evade immune-mediated elimination of immunogenic tumor cells. The costimulatory signals generated by myeloid tumor cells can induce T cell “exhaustion” through the upregulation of immune checkpoint proteins, such as PD-1, LAG3, and TIM3, thereby resulting in the development of advanced immune escape strategies [17]. The exhausted T cells present decreased production of cytokines and gradual loss of killing function and promote the immune escape of malignant cells. T cell exhaustion and myeloid blast proliferation form a vicious cycle in patients with MDS, leading to the progression of the disease. A significant number of mutations were observed in the checkpoint genes in patients with MDS [18]. Blockade of immune checkpoint proteins partially restores T cell function in patients with malignant tumor or mice with tumor [19, 20]. Accumulating evidence supports the therapeutic potential of targeting exhausted T cells. Cellular immunotherapy through immune checkpoint inhibition may provide a novel treatment idea for patients with MDS. Thus, a deeper understanding of the mechanism of T cell exhaustion is crucial for establishing rational immunotherapy interventions. LAG3 is one of the immune checkpoint proteins, and it is a negative regulator of effector T cell both in vitro and in vivo. After the binding of LAG3 to its ligand, CD3/T cell receptor (TCR) is downregulated and calcium influx is restricted, leading to the inhibition of T cell function [21]. LAG3 also prevents T cells from entering the growth phase beyond the S phase of the cell cycle, thus inhibiting the activation and amplification of effector T cells [22]. SNPs in LAG3 have been found to be involved in various malignant diseases. For example, SNPs in the intron region of *LAG3* (rs2365094 and rs3782735) have been found to be significantly associated with susceptibility to multiple myelomas [23]. In our previous study, we found that the expression of LAG3 is upregulated in patients with MDS. In this study, we found that the frequency of rs1941928301 in the intronic region of *LAG3* is significantly higher in patients with MDS than in healthy controls. There may be correlations between the two. Although introns are not involved in the final protein coding, studies have shown that introns can increase the rate of transcription, nuclear export, transcript stability, and translation efficiency to achieve intron-mediated increase in gene expression [24], and their role in the regulation of gene expression cannot be ignored. Rs1941928301 (C>T) in the intron of *LAG3* may be one of the factors involved in high LAG3 expression in patients with MDS that leads to inhibited T-cell function and myeloid clone amplification. However, evidence of functional significance of this LAG3 SNP is not yet available. Additional functional biological studies are needed to explore the mechanisms in which the genetic variants may influence the expression of LAG3 and the pathogenesis of MDS.

SNPs are the most common type of genetic alterations that lead to disease susceptibility in autoimmune diseases. The SNPs associated with IRP have not been studied to date. In our study, the frequency of the six SNPs found in patients with IRP showed no significant difference from that in healthy individuals. Various SNPs, such as HLA-DPB1 rs1042151, perforin gene rs885822, and interferon-gamma+874, have been found in patients with SAA. In our study, three SNPs at position 6883700(rs2365095), 6886722(rs199912414), and 6887020(rs870849) were observed in patients with SAA. Furthermore, there was no significant difference in the level of allelic and genotypic frequency of the SNPs within *LAG3* between patients with SAA and healthy controls. We concluded that the investigated SNPs in *LAG3* were not significantly associated with SAA or IRP risk.

## 5. Conclusion

In conclusion, our results indicate that LAG3 rs1941928301 might be associated with a higher risk of MDS. The detected LAG3 SNPs have no apparent effect on susceptibility to SAA and IRP. This study does have some limitations. We need to increase the number of patients to further confirm the association. However, we did find an SNP in patients with MDS whose incidence was different from that in healthy controls, and this SNP may be one of the genetic predisposition factors of some patients with MDS. This difference in SNP among patients with different bone marrow diseases might be useful in the differential diagnosis of MDS and SAA. It might also lead to improved protocols for the treatment of bone marrow failure diseases.

## Figures and Tables

**Table 1:** Characteristics of all study participants.

Characteristics	SAA (*n* = 16)	IRP (*n* = 25)	MDS (*n* = 21)	HC (*n* = 16)	*P*
Median age (years, range)	49 (19–68)	48 (11–71)	56 (29–72)	41.5 (24–62)	>0.05
Sex					>0.05
Male, no. (%)	9 (56.3)	12 (48.0)	16 (76.2)	6 (37.5)	
Female, no. (%)	7 (43.7)	13 (52.0)	5 (23.8)	10 (62.5)	
Blood routine test					
RBC (×10^12^/L)	2.10 ± 0.34	2.57 ± 0.58	2.34 ± 0.93	4.27 ± 0.46	
Hb (g/L)	68.38 ± 11.36	85.16 ± 18.61	75.48 ± 25.39	134.06 ± 12.99	
WBC (×10^9^/L)	1.56 ± 0.78	3.64 ± 1.76	3.49 ± 2.83	5.16 ± 1.40	
N (×10^9^/L)	0.30 ± 0.23	1.79 ± 1.39	1.68 ± 1.84	2.49 ± 0.63	
L (×10^9^/L)	1.16 ± 0.60	1.55 ± 1.08	1.20 ± 0.60	2.20 ± 1.54	
M (×10^9^/L)	0.10 ± 0.07	0.25 ± 0.12	0.55 ± 0.84	0.41 ± 0.15	
PLT (×10^9^/L)	20.56 ± 16.13	42.4 ± 43.51	71.81 ± 88.49	201.00 ± 102.14	
Ret (×10^9^/L)	18.40 ± 15.65	51.66 ± 28.70	50.51 ± 41.91	65.98 ± 13.65	

Notes: SAA: severe aplastic anemia; IRP: immune-related pancytopenia; MDS: myelodysplastic syndrome; HC: healthy control; RBC: red blood cell; Hb: hemoglobin; WBC: white blood cell; N: neutrophil; L: lymphocyte; M: monocyte; PLT: platelet; Ret: reticulocyte.

**Table 2:** Sequences of amplified primers and sequencing primers.

No.	Name of the amplified primer	Amplified primer sequence	Name of sequencing primer	Sequencing primer sequence
1	LAG3-Exon1-F1	GAGACCCAGGACTGAGGAAGTAA	LAG3-Exon1-seqF	ACCACAGAGGTGGAGAGGTG
	LAG3-Exon1-R1	CACAGAGCTAAGGACCTCAGGA		
2	LAG3-Exon2-F1	CCTGAGGTCCTTAGCTCTGTGG	LAG3-Exon2-seqF	GGGCAGAGAAGAAACAGAAACC
	LAG3-Exon2-R1	GACTGGGTCACTGGCTTCAGA		
3	LAG3-Exon3-F1	CCTTCTCTCCAGAAGTGGATGC	LAG3-Exon3-seqF	GGACGGTTGGTGGTCAAGAG
	LAG3-Exon3-R1	CACCCCCTTCCTCTACTCTTCT		
4	LAG3-Exon4-F1	GTCTTGGGGATCCACTTTATGC	LAG3-Exon4-seqF	GTCTTGGGGATCCACTTTATGC
	LAG3-Exon4-R1	GGTCAAATGTGAAGCAGAGTGG		
5	LAG3-Exon5-F1	ATCACCCCTTCTTGCTTCTCC	LAG3-Exon5-seqF	AGCCTCCTCAGCTCATCACC
	LAG3-Exon5-R1	CAGCTACTCCTTTCCCACCTGA		
6	LAG3-Exon6-Exon7-F1	CAAGTGAGTGCAGGGTGATTG	LAG3- Exon7-seqF	CAAACGCCACAGCAATAATCTT
	LAG3-Exon6-Exon7-R1	AGCACTCTGAGGATGGAAGAGC	LAG3-Exon6-seqR	TACAGGATGAGGCATAGGGTCA
7	LAG3-Exon8-F1	GATGGCTTGTGCCTGGGTAG	LAG3-Exon8-seqF	GATGGCTTGTGCCTGGGTAG
	LAG3-Exon8-R1	GAAAGAGAGAGTGAGCGCAAGA		

LAG3: lymphocyte activation gene 3.

**Table 3:** SNPs in all study participants.

No.	Position	Ref	Alt	Fun	Frequency of minor allele			
					MDS	SAA	IRP	HC
1	6881748	G	A	UTR5	0	0	0.02	0
2	6881835	G	A	UTR5	0	0	0.04	0.0625
3	6881946	C	A	UTR5	0.02381	0	0	0
4	6883680	A	T	Intronic	0.02381	0	0.02	0
5	6883700	C	T	Intronic	0.2857	0.25	0.16	0.1875
6	6884554	T	C	Exonic	0	0	0	0.03125
7	6886722	A	G	Intronic	0	0.03125	0	0
8	6887020	T	C	Exonic	0.1429	0.2812	0.26	0.15625
9	6887343	C	T	Intronic	0.119	0	0.04	0
10	6887348	C	G	Intronic	0.04762	0	0	0
11	6887384	G	C	Intronic	0.04762	0	0	0

Notes: SNP: single-nucleotide polymorphism; Ref: reference allele; Alt: altered allele; Fun: function of reference genotype; MDS: myelodysplastic syndrome; SAA: severe aplastic anemia; IRP: immune-related pancytopenia; HC: healthy control.

## Data Availability

The data that support the findings of this study are available from the corresponding author upon reasonable request.
